# Molecular Basis of Selectivity and Activity for the Antimicrobial Peptide Lynronne‐1 Informs Rational Design of Peptide with Improved Activity

**DOI:** 10.1002/cbic.202100151

**Published:** 2021-06-08

**Authors:** Eleanor S. Jayawant, Jack Hutchinson, Dorota Gašparíková, Christine Lockey, Lidón Pruñonosa Lara, Ciaran Guy, Rhiannon L. Brooks, Ann M. Dixon

**Affiliations:** ^1^ Department of Chemistry University of Warwick Gibbett Hill Road Coventry CV4 7AL UK; ^2^ SynBio Doctoral Training Centre University of Warwick Gibbet Hill Road Coventry CV4 7AL UK; ^3^ MAS Centre for Doctoral Training University of Warwick Gibbet Hill Road Coventry CV4 7AL UK

**Keywords:** Helical antimicrobial peptide, model membranes, NMR spectroscopy, peptide engineering, structure activity relationship

## Abstract

Antibiotic resistance is a significant threat to human health, with natural products remaining the best source for new antimicrobial compounds. Antimicrobial peptides (AMPs) are natural products with great potential for clinical use as they are small, amenable to customization, and show broad‐spectrum activities. Lynronne‐1 is a promising AMP identified in the rumen microbiome that shows broad‐spectrum activity against pathogens such as methicillin‐resistant *Staphylococcus aureus* and *Acinetobacter baumannii*. Here we investigated the structure of Lynronne‐1 using solution NMR spectroscopy and identified a 13‐residue amphipathic helix containing all six cationic residues. We used biophysical approaches to observe folding, membrane partitioning and membrane lysis selective to the presence of anionic lipids. We translated our understanding of Lynronne‐1 structure to design peptides which varied in the size of their hydrophobic helical face. These peptides displayed the predicted continuum of membrane‐lysis activities *in vitro* and *in vivo*, and yielded a new AMP with 4‐fold improved activity against *A. baumannii* and 32‐fold improved activity against *S. aureus*.

## Graphical abstract

**Here we describe** the biophysical characterization of Lynronne‐1, an antimicrobial peptide found in the bovine rumen microbiome. We reveal the location of an amphipathic helix containing all six cationic residues, and observe lipid‐selective folding, membrane‐binding and lysis. Our structural data were used to design three variants with changes in the size of the hydrophobic helical face, one of which yielded 32‐fold improved activity *in vivo*.

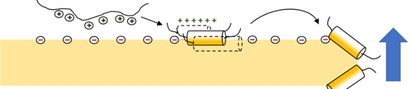

## Introduction

Antibiotic resistance has become a significant threat to human health, in part due to the overuse and misuse of antibiotics in both humans and livestock. In an effort to counter this impending resistance crisis, a concerted effort to identify and develop new antimicrobials has emerged. This effort is largely focused on natural products, which have yielded many of the most successful antibiotics known, such as penicillins^[1]^ and cephalosporins,^[2]^ and remain one of the best sources for new compounds.^[3]^ A structurally diverse range of naturally‐occurring antimicrobials can be found, from small organic molecules like macrolides, to vast protein assemblies such as pyocins.^[4]^ Antimicrobial peptides (AMPs) are a further class of natural product with great potential for clinical use.[^5^, ^6^, ^7^] AMPs are small enough to be produced and administered with relative ease, highly amenable to customization via sequence modifications, and offer advantages such as broad‐spectrum antimicrobial activity as well as antifungal, antiviral, antiparasitic and anticancer properties. Unlike most small molecule antibiotics, one of the mechanisms of action for AMPs is the disruption/ lysis of bacterial membranes. These membranes are evolutionarily conserved components of bacterial cells and define the phenotype. Therefore, it is expected that bacterial resistance will develop more slowly against AMPs, because alterations of the membrane would be costly for the bacteria.^[8]^

Although some AMPs are already in clinical use, such as polymyxins and gramicidin S for treatment of *P. aeruginosa* and *Acinetobacter* infections,[^9^, ^10^] a generation of new AMPs is necessary to address issues of high toxicity in eukaryotic cells and susceptibility to proteolytic degradation. One innovative method for discovery of new AMPs is “mining” of DNA from organisms that produce defence compounds when confronted by competing bacteria. The Lynronne‐1 peptide was identified by Oyama et al.^[11]^ using a metagenomics and computational approach to investigate the bovine rumen microbiome for the presence of novel AMPs. In this study multiple candidate AMPs were identified, the most promising of which was the 19 amino acid cationic peptide Lynronne‐1. Lynronne‐1 was shown to have broad‐spectrum activity against a number of common gram‐positive and gram‐negative pathogenic bacteria including methicillin‐resistant *Staphylococcus aureus* (MRSA), where Lynronne‐1 yielded an MIC of 8–32 μg/mL within the range of most commercially available AMPs, and *Acinetobacter baumannii* where an MIC of 4 μg/mL was achieved across five different strains. Although its *in vivo* activity is lower than conventional antibiotics, Lynronne‐1 was shown to have a greater selectivity for bacterial cells, exhibiting low haemolytic activity against blood cells and low cytotoxicity against mammalian cells. Lynronne‐1 demonstrated faster activity against three MRSA strains than vancomycin, a commercial glycopeptide antibiotic used to treat MRSA. A rapid mode of action is considered a strong indicator that the rate of resistance developed by targeted pathogens is low.^[12]^ Lynronne‐1 is also highly active against *Acinetobacter baumannii*, an ESKAPE pathogen resistant to a wide range of antibiotics and with an increasing incidence of hospital‐derived infection. A systematic review of antibiotic resistance showed that ESKAPE pathogens are associated with the highest risk of mortality, and that antibiotic‐resistant ESKAPE bacteria result in significantly increased healthcare costs.^[13]^ In particular, carbapenem‐resistant *A. baumannii* was recently listed by the World Health Organization (WHO) as a pathogen of critical priority for which new antibiotics are urgently needed,^[14]^ and pan‐resistance (resistance to all known classes of antibiotics) has also been observed for *A. baumannii*,^[15]^ highlighting the need for new treatment strategies against this pathogen.

**Figure 1:**
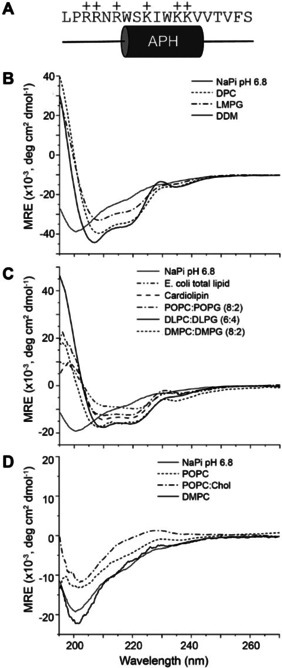
A. Primary sequence of the Lynronne‐1 antimicrobial peptide and the predicted location of the amphipathic helix (APH) from previous studies. Circular dichroism spectra of Lynronne‐1 in B. detergent micelles, C. “bacterial” model membranes, and D. “mammalian” model membranes. All data were measured at 37 °C and are given in units of mean residue ellipticity (MRE).

The favourable activity and high specificity of the Lynronne‐1 peptide make understanding its structure and mode of action an area of keen interest. Thus far, there are no high‐resolution structural data for Lynronne‐1, although computational models of the peptide were created using the programme Pep‐Fold^[16]^ and these indicated a section of α‐helical structure (Figure 1A) with an amphipathic nature. Amphipathic helices are a common motif among antimicrobial peptides, as is a net positive charge due to enrichment of cationic residues (Lynronne‐1 has a net positive charge of +6). Amphipathic helices are particularly well‐known to associate with bilayer membranes, leading to membrane permeabilization and lysis according to established models such as the carpet model,^[17]^ the barrel‐stave model, and the toroidal pore model.^[18]^ Indeed, it was suggested by Oyama and co‐workers^[11]^ that Lynronne‐1 activity is due to pore formation in bacterial cell membranes. It was also shown that Lynronne‐1 preferentially inserted into lipid monolayers containing lipids specific to bacteria, such as 1‐palmitoyl‐2‐oleoyl‐*sn*‐glycero‐3‐(phospho‐*rac*‐(1‐glycerol)) (POPG), cardiolipin (CL), lipoteichoic acid (LTA) and phosphatidylethanolamine (POPE). Such specificity would explain its low cytotoxicity and broad‐spectrum activity.

While these results are excellent indicators of the mode of action, they cannot distinguish between the various models of membrane activity nor do they reveal the structure of Lynronne‐1, which is required to develop a model of its function. We have investigated the first stages of membrane binding by this promising AMP in order to develop a model of its structure, lipid selectivity and interactions both in aqueous solution and in the presence of lipid bilayers of varying composition. Using a variety of biophysical methods, including solution‐state NMR spectroscopy, we assess secondary structure, membrane partitioning, and membrane lysis. Measurements were made *in vitro* using model membranes with compositions reflective of mammalian and bacterial membranes in order to investigate the predominant source of lipid selectivity observed in this peptide. Finally, we used our new understanding of Lynronne‐1 structure to rationally design a series of peptides predicted to display a continuum of membrane‐lysis activities. The approaches and results reported here yield a unified model of Lynronne‐1 activity and directed the engineering and rational modification of a new AMP with four‐fold improved activity against *A. baumannii* and 32‐fold improved activity against *S. aureus*.

## Results and Discussion

### Lynronne‐1 binding to membranes is highly lipid‐dependent and results in a helical structure

The Lynronne‐1 peptide was predicted by Oyama et al.^[11]^ to fold into a structure possessing an 8‐residue amphipathic α‐helix (residues W_7_‐V_14_) that directs binding to bacterial cell membranes. However, the structure of Lynronne‐1 has not yet been determined experimentally. To investigate secondary structure, circular dichroism (CD) was used to analyse a 19‐residue peptide corresponding to Lynronne‐1 (Figure 1A) in a range of solution environments. A library of model membranes, varying in their lipid composition to represent both mammalian and bacterial cell membranes as summarized in Table 1, were prepared as small unilamellar vesicles (SUVs). Model bacterial membranes were prepared from cardiolipin, to support results published in Oyama et al.^[11]^ for lipid monolayers, and *E. coli* total lipid extract. SUVs were also prepared from phosphatidylcholine (PC) and phosphoglycerol (PG) lipids with a variety of chain lengths and saturation, to explore the requirements for anionic lipids. Model mammalian membranes were prepared, in which anionic PG lipids were omitted or replaced with the common mammalian membrane component cholesterol. Finally, Lynronne‐1 folding was evaluated in *n*‐dodecylphosphocholine (DPC), 1‐myristoyl‐2‐hydroxy‐sn‐glycero‐3‐phospho‐(1′‐rac‐glycerol) (LMPG), and *n*‐dodecyl‐*b*‐D‐maltopyranoside (DDM) detergent micelles. While micelles are less optimal mimetics of cell membranes, their use facilitated high‐resolution structure characterization of this peptide as has been demonstrated for other well‐known AMPs such as Magainin,^[19]^ Melittin,^[20]^ and Gramicidin.^[21]^

**Table 1:** Model membranes used in this work, highlighting head group identity and net charge. Also shown is the wavelength of maximum emission (λ_em_) for the tryptophan residue under each set of conditions, as well as the change (shift) in wavelength upon exposure to each model membrane.

	Lipid composition	Head group	Charge	Tryp. λ_em_ [nm]	Shift [nm]
Bacterial	*E. coli* total	PE:PG:CL	Anionic	335	−22
	Cardiolipin	CL	Anionic	332	−25
	POPC:POPG (8 : 2)	PC:PG	Anionic	334	−23
	DLPC:DLPG (6 : 4)	PC:PG	Anionic	332	−25
	DMPC:DMPG (8 : 2)	PC:PG	Anionic	336	−21
Mammalian	POPC	PC	Zwitterionic	357	0
	DMPC	PC	Zwitterionic	352	−5
	POPC:Chol. (8 : 2)	PC	Zwitterionic	357	0
Detergent	DPC	PC	Zwitterionic	339	−18
	LMPG	PG	Anionic	333	−24
	DDM	M	Uncharged	339	−18
Buffer	25 mM NaPi pH 6.8	n/a	n/a	357

Figure 1B shows the CD spectrum for Lynronne‐1 in aqueous buffer solution (25 mM NaPi, pH 6.8). This spectrum is characteristic of a random coil peptide structure with a negative minimum at ∼200 nm and no other strongly defining features. In the presence of detergent micelles, at a 1 : 1 ratio of micelle to peptide, the spectra displayed features of an α‐helical fold, containing negative peaks at 208 and 222 nm and a positive peak at ∼195 nm. High helical content was also observed in CD spectra of Lynronne‐1 in the presence of our bacterial model membranes (Figure 1C). Conversely, in the presence of the mammalian model membranes containing only zwitterionic components, very little change from the unfolded aqueous spectrum was observed (Figure 1D).

**Figure 2:**
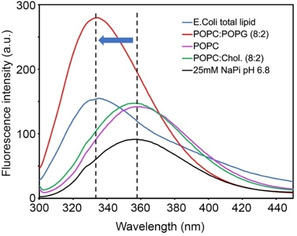
Tryptophan fluorescence emission spectra of Lynronne‐1 in the presence of representative mammalian and bacterial model membranes. Data were collected using an excitation wavelength of 280 nm to excite the two central Trp residues in Lynronne‐1. A blue shift (indicated by an arrow) was observed for the Trp emission peak in the presence of the bacterial membrane mimetics, but no blue shift was observed in buffer or the mammalian mimetics.

The partitioning of Lynronne‐1 to our library of model membranes was studied using intrinsic tryptophan fluorescence, a method that does not require a change in fold to report peptide‐bilayer interactions. This method relies on a shift in the tryptophan emission wavelength with a change in the polarity of the local environment, as would be the case upon partitioning of a peptide from aqueous solution to a hydrophobic membrane. Figure 2 shows representative Trp emission spectra for Lynronne‐1 in model membrane bilayers, where a blue shift in Trp emission from 357 nm in aqueous buffer (black spectrum) to 332–336 nm in bacterial model membranes (red and blue spectra) is observed. A similar blue shift is observed in all detergent micelles, with the largest shift in the anionic detergent LMPG (see Table 1). Conversely, a blue shift is not observed in mammalian model membranes (green and magenta spectra). The wavelengths of maximum Trp emission (λ_em_) for Lynronne‐1 in each model membrane are given in Table 1 and indicate that the peptide selectively associates with detergent micelles and bacterial (anionic) model membranes, while no association is observed with mammalian model membranes.

### Structural characterization of Lynronne‐1 by solution state NMR spectroscopy

**Figure 3:**
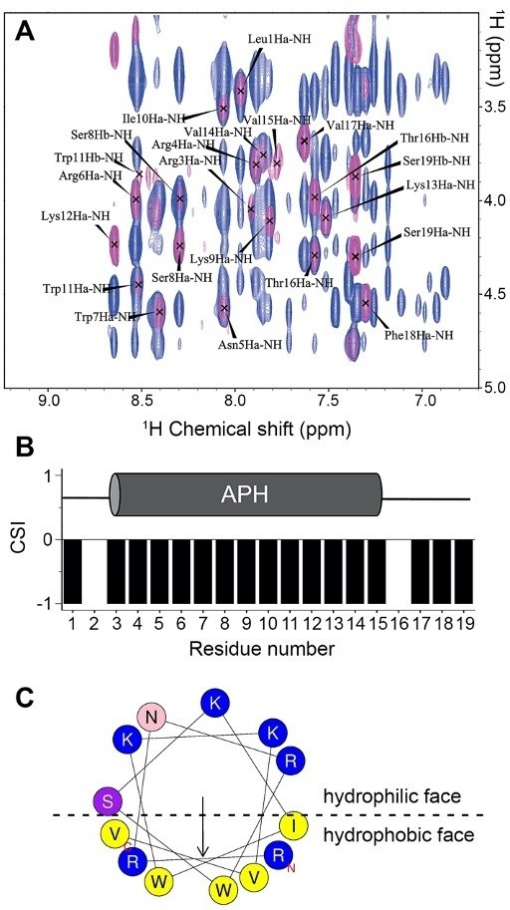
A. Overlay of ^1^H‐^1^H NOESY (τ_m_=150 ms, blue spectrum) and ^1^H‐^1^H TOCSY (τ_m_=100 ms, pink spectrum) showing the fingerprint region of Lynronne‐1 (0.6 mM) in 25 mM sodium phosphate buffer, pH 6.8, containing 100 mM DPC‐*d_38_*. The Hα‐NH correlations required to perform a backbone‐walk have been labelled. B. Plot of Hα chemical shift index (CSI) versus residue number for Lynronne‐1 in DPC‐*d_38_*. A schematic of the helical region is shown above the plot. C. Helical wheel plot of the NMR‐derived helical region in Lynronne‐1. Cationic residues are displayed in blue, polar uncharged residues are shown in pink and purple, and hydrophobic residues are displayed in yellow.

The location of the helical region within Lynronne‐1 was determined using two‐dimensional homonuclear solution state NMR. Sequential assignment of ^1^H nuclei in the peptide was achieved using ^1^H‐^1^H TOCSY and ^1^H‐^1^H NOESY spectra of the peptide solubilized in DPC micelles, a membrane mimetic which yielded a highly helical structure (Figure 1B) and is readily available in fully deuterated form. Figure 3A shows an overlay of the fingerprint region of Lynronne‐1 TOCSY and NOESY spectra, with residue assignments indicated. The full ^1^H assignment for Lynronne‐1 is given in Table S1, in which 88 % of all ^1^Hs were assigned, and the assignments were used to perform a ^1^Hα chemical shift index (CSI) analysis^[22]^ (Figure 3B). The NMR data in the presence of DPC were indicative of α‐helix formation between residues R_3_‐V_15_, a region that is substantially larger than previously predicted. This region is plotted on a helical wheel representation in Figure 3C where the amphipathic nature of the helix can be clearly visualised. This arrangement of hydrophobic and hydrophilic residues on separate helical faces is a trademark of antimicrobial peptides.

### Lynronne‐1 preferentially disrupts membranes composed of anionic lipids

**Figure 4:**
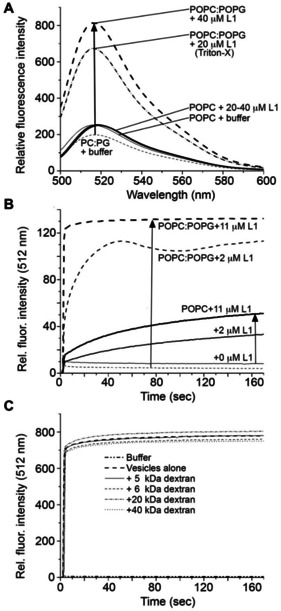
A. Steady‐state fluorescence spectra of carboxyfluorescein‐loaded vesicles, prepared from either POPC (solid lines) or 8 : 2 POPC:POPG (dashed lines) lipids, after addition of 25 mM sodium phosphate buffer or buffer containing increasing concentrations of Lynronne‐1 (L1). Triton X (10 %) detergent was added to induce full leakage, and yielded a spectrum that overlaps strongly with the data collected for POPC:POPG with 20 μM Lynronne‐1. B. Fluorescence time course measurements collected at 512 nm for carboxyfluorescein‐loaded POPC (solid lines) and 8 : 2 POPC:POPG (dashed lines) vesicles in the absence and presence of increasing concentrations of Lynronne‐1. C. Fluorescence time course measurements collected at 512 nm for carboxyfluorescein‐loaded 8 : 2 POPC:POPG vesicles in the presence of 5 μM Lynronne‐1 and dextran (20 μM) at a range of molecular weights.

Early work by Oyama et al.^[11]^ suggested that Lynronne‐1 preferentially binds to bacterial membranes, and induces membrane permeability, leading to cytoplasmic leakage. In order to investigate this further, an *in vitro* vesicle disruption assay was conducted. Briefly, SUVs were loaded with the fluorescent dye carboxyfluorescein at self‐quenching concentrations as described in the Materials and Methods and in detail by Jimah and co‐workers.^[23]^ Carboxyfluorescein fluorescence was monitored in SUVs composed of either POPC or POPC:POPG (8 : 2) using an excitation wavelength of 492 nm, and emission was measured between 500–600 nm (Figure 4A). In the absence of Lynronne‐1, both SUV types yielded comparable emission spectra with similar emission wavelengths and intensities (indicating equivalent vesicle and fluorophore concentrations). Each SUV type was then treated with two different concentrations of Lynronne‐1 for 30 seconds, and the resultant fluorescence intensity measured. While very little change in the fluorescence emission spectrum was observed for the POPC bilayers, addition of Lynronne‐1 to the POPC:POPG bilayers resulted in a large increase in fluorescence. This increase indicates release of the dye into bulk solution, where it is diluted to a concentration below the self‐quenching concentration, and is a clear demonstration of membrane disruption and lysis^[23]^ in a dose‐dependent manner.

Carboxyfluorescein fluorescence emission at 512 nm was measured over time in both model membrane systems after the addition of peptide to a final concentration of either 2.3 or 11.3 μM. Figure 4B shows the fluorescence intensity, compared to the value obtained upon addition of Triton‐X representing maximum lysis, over time. For the mammalian model membrane (POPC), there was a slow but steady increase in fluorescence intensity upon the addition of Lynronne‐1 that did not reach saturation over the time period monitored. In the bacterial model membrane (POPC:POPG (8 : 2)), there was an almost instant increase in emission intensity upon addition of 11.3 μM peptide which reached saturation in a matter of seconds. These data suggest that while Lynronne‐1 is weakly lytic on zwitterionic membrane surfaces, the interaction with anionic PG headgroups induces rapid membrane disruption and subsequent lysis and is in keeping with the rapid kill kinetics reported by Oyama et al.^[11]^

To determine if Lynronne‐1‐derived membrane permeability occurs via discrete pore formation,^[18]^ as opposed to formation of transient holes via the carpet/detergent‐like mechanism,^[17]^ dextran molecules of varying sizes were included in the lysis assay. This approach is built on the premise that appropriately sized dextran molecules will block discrete pores and inhibit release of carboxyfluorescein,^[23]^ and has been used successfully in the past to confirm the pore‐forming model of action for a malaria vaccine candidate.^[24]^ Figure 4C shows time‐course emission spectra in samples containing POPC:POPG (8 : 2) vesicles, Lynronne‐1 (5 μM), and a molar excess of dextran molecules (20 μM) of varying molecular weights. A small degree of variation was observed in these spectra when dextrans were present (2–4 % change in the maximum fluorescence intensity), however the magnitude of this effect suggests that any impact of dextrans is largely non‐specific, and could result from excess dextran inhibiting interactions between Lynronne‐1 and the vesicle surface. The results of the dextran‐based approach suggest that discrete Lynronne‐1 pores do not form, however we acknowledge potential limitations of this method. The size of dextran molecules investigated may be a poor match for the pores formed. The presence of dextran may also impair formation of these pores. Therefore, while we accept that these data do not exclude the presence of a pore, they suggest that Lynronne‐1 acts on the membrane via an alternative mechanism less likely to be affected by dextran, such as the carpet/detergent model. Adding weight to this hypothesis is the fact that the APH formed by Lynronne‐1 is only 13 residues in length and thus predicted to extend only 19.5 Å (5.4 Å per 3.6 helical residues). Compared with the reported hydrophobic thickness of a POPC bilayer (27.1 Å)^[25]^ and a POPG bilayer (27.8 Å),^[26]^ this APH is too short to stably span the membrane. Indeed, recent work has demonstrated that length is a crucial feature for pore‐forming helical AMPs.^[27]^

### Rational modifications to the hydrophobic helical face of Lynronne‐1 lead to predictable changes in membrane lysis activity

**Figure 5:**
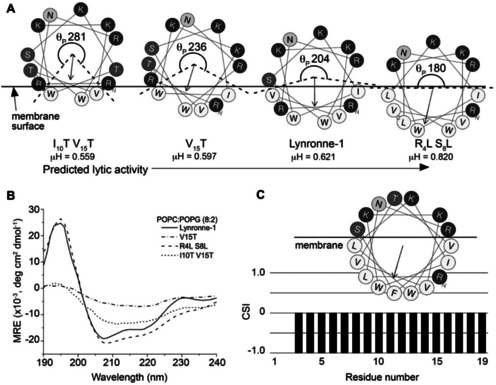
A. Rational design of three Lynronne‐1 variants predicted to have decreasing (V_15_T, I_10_T‐V_15_T) and increasing (R_4_L‐S_8_L) lytic activities compared to Lynronne‐1 by varying the size of the hydrophobic face (as evidenced by the hydrophobic moment μH) and polar angle (θ_P_) in the amphipathic helix. B. CD spectra of Lynronne‐1 and variants (0.1 mg/mL) dissolved in 25 mM sodium phosphate buffer (pH 6.8) containing 1.2 mg/mL of POPC:POPG (8 : 2) vesicles. **C**. Plot of the Hα chemical shift index (CSI) versus residue number for the R_4_L‐S_8_L peptide (0.8 mM) in DPC‐*d_38_*. Chemical shifts were obtained from ^1^H‐^1^H NOESY and TOCSY spectra (see representative spectra in Figure S5). A helical wheel diagram of the NMR‐derived helical region in the R_4_L‐S_8_L peptide is shown above the CSI plot.

The results above lend themselves to a model for Lynronne‐1 activity in which the AMP is unstructured in aqueous solution, and transitions to an α‐helical structure upon binding selectively to membranes containing anionic lipids. The 13‐residue helical region of Lynronne‐1 stretches from R_3_‐V_15_ and forms an amphipathic helix, which contains all six of the basic residues in Lynronne‐1, that rapidly lyses membranes containing anionic lipids such as PG. Using this model as a foundation, we explored the impact of small alterations in the Lynronne‐1 sequence on its ability to lyse membranes. Specifically, we prepared a series of variants in which the size of the hydrophobic helical face was systematically increased or decreased as shown in Figure 5A. The size of the hydrophobic face is inversely proportional to the polar angle, θ_P_,^[28]^ or the angle delineating the polar face of the helix, and this angle has also been shown in Figure 5A. One variant was prepared in which the size of the hydrophobic face was increased (and corresponding θ_P_ decreased) by substituting polar Arg and Ser residues in positions 4 and 8 with Leu residues to create the R_4_L S_8_L peptide. This peptide has a higher hydrophobic moment (μH) than Lynronne‐1 and we predicted that it would have increased lytic activity compared to wild‐type due to the deeper penetration depth of the APH into the bilayer. Likewise, two additional variants were created in which the sizes of the hydrophobic face and resulting hydrophobic moments were systematically decreased (while θ_P_ was increased) by substituting hydrophobic residues with polar Thr residues yielding V_15_T and I_10_T V_15_T peptides predicted to display decreasing lytic activities. Thr was selected to introduce polarity without introducing any further charge.

The secondary structures of each of these peptides were measured using CD for samples containing 50 uM peptide and 1.2 mg/mL POPC:POPG (8 : 2) vesicles, and compared to an equivalent spectrum for wild‐type Lynronne‐1 in Figure 5B. A reduction in α‐helicity was observed for the V_15_T and I_10_T V_15_T peptides when compared to Lynronne‐1. Conversely, the R_4_L S_8_L peptide appeared to have an increased helical content under the same conditions, as indicated by the increased intensities of peaks at 195, 208 and 222 nm. The location of the helical region in the R_4_L S_8_L peptide was mapped as described for Lynronne‐1, using 2D ^1^H‐^1^H TOCSY and NOESY data. The spectra and full assignments are given in Figures S5 and Table S2, respectively, and CSI analysis of Hα chemical shifts (Figure 5C) was used to identify a helical region from R_3_‐S_19_ which is highly amphipathic with a large and well‐defined hydrophobic face (Figure 5C). The lytic activity of each peptide was then measured using time‐course fluorescence emission spectra of carboxyfluorescein‐loaded POPC:POPG (8 : 2) vesicles, after addition of peptide to final concentrations of 11, 15 and 20 μM (Figure S6). The observed trend in lytic activity was identical at all concentrations, specifically R_4_L S_8_L>Lynronne‐1=V_15_T>I_10_T V_15_T, indicating that the size of the hydrophobic helical face in this sequence correlates strongly with ability to lyse model bacterial membranes.

### Antimicrobial activity of Lynronne‐1 derivatives correlates with predictions from structure

Lynronne‐1 was shown previously to have antimicrobial activity against both *A. baumannii* and *S. aureus*, with reported minimum inhibitor concentrations (MICs) of 8–32 μg/mL for *S. aureus* and 4 μg/mL *A. baumannii*.^[11]^ In this work, we screen antimicrobial activity of Lynronne‐1 against these two organisms and compared the activity to those measured for our three designed variants as well as a positive control, carbenicillin (expected MIC of 2–8 μg/mL in *S. aureus* ATCC 29213). The resulting MICs are given in Table 2. The MICs for Lynronne‐1 we obtained were higher than those obtained in the previous study: we observed MICs no lower than 16 μg/mL for either organism. This increase is likely due to differences in the materials used, and specifically on the chemical composition of the microplates. Oyama et al.^[11]^ used polypropylene microplates in their study, while we were unable to source these plates and instead used polystyrene microplates. Given that polystyrene plates have been shown to alter the MIC of cationic antimicrobial peptides in the past,^[29]^ it is highly likely that this is the source of the discrepancy. We sought to minimise the impact of polystyrene binding by preparing all peptide solutions in low‐binding polypropylene Eppendorf tubes.

**Table 2:** Minimum inhibitory concentrations (MIC) of Lynronne‐1 and derivatives. Data includes three separate biological repeats (log2 standard deviation (SD) is indicated in parentheses, 2 sf). For I10T V15T, SD was not determined (ND) as it was reproducibly inactive (MIC>256 μg/mL).

Strain	MIC [μg/mL]				
	Carb.	WT	R4L S8L	V15T	I10T V15T
*A. baumannii* ATCC 19606	16–8 (0.47)	32–16 (0.47)	8 (0)	16 (0)	>256–128 (ND)
*S. aureus* ATCC 29213	4 (0)	256–64 (0.94)	8 (0)	256–128 (0.47)	>256 (ND)
*S. aureus* ATCC 6538	2–0.5 (0.82)	64–16 (0.82)	8–1 (1.25)	256–64 (0.94)	>256 (ND)

The focus of the antimicrobial screening was to confirm whether our designed variants had the predicted impact on antimicrobial activity *in vivo*. All three variants were screened against select strains of *A. baumannii* and *S. aureus*, and the resulting MICs are given in Table 2. The I_10_T V_15_T peptide, predicted to be the least active of the peptides, yielded the highest MICs (typically>256 μg/mL) for both organisms. The V_15_T variant had activities which were similar to those observed for Lynronne‐1, or intermediate between Lynronne‐1 and I_10_T V_15_T. Finally, we observed that the R_4_L S_8_L peptide, predicted to be the most active of the four peptides tested, yielded up to four‐fold increased antimicrobial activity against *A. baumannii* (i. e. 8 vs. 16–32 μg/mL) and up to 32‐fold increased antimicrobial activity against *S. aureus* (1–8 vs. 16–256 μg/mL) when compared to Lynronne‐1. These results suggest that our strategy for rational modification of the size of the hydrophobic helical face in Lynronne‐1 results in increased antimicrobial activity. These results also suggest that the biophysical data in model membranes is highly reflective of the behaviour of this peptide in cells. Overall, R_4_L S_8_L shows promising activity that is comparable to or better than the control antibiotic carbenicillin in two of the three strains screened.

While this increased antimicrobial activity was encouraging, it was necessary to assess the haemolytic activity of the peptides to detect any potential non‐specific lysis of mammalian cells. It has been shown that excessive hydrophobicity in other AMPs has resulted in loss of selectivity and increased erythrocyte lysis.^[30]^ As shown in Table 3, V_15_T and I_10_T V_15_T demonstrated haemolytic activity against erythrocytes only at very high concentrations (>970 μg/mL). This may reflect their lower biological activity in general, rather than a low specificity for mammalian cells. Treatment of erythrocytes with Lynronne‐1 and R_4_L S_8_L resulted in haemolysis at lower concentrations (536 and 164 μg/mL, respectively), however for R_4_L S_8_L this concentration is still >20 times higher than the MIC for all strains screened, suggesting that R_4_L S_8_L has great potential as a therapeutic.

**Table 3:** Haemolytic activity of Lynronne‐1 and variants.

Maximum concentration resulting in no haemolysis [μg/mL]			
WT	R4L S8L	V15T	I10T V15T
536	164	>1200	970

## Conclusion

**Figure 6:**
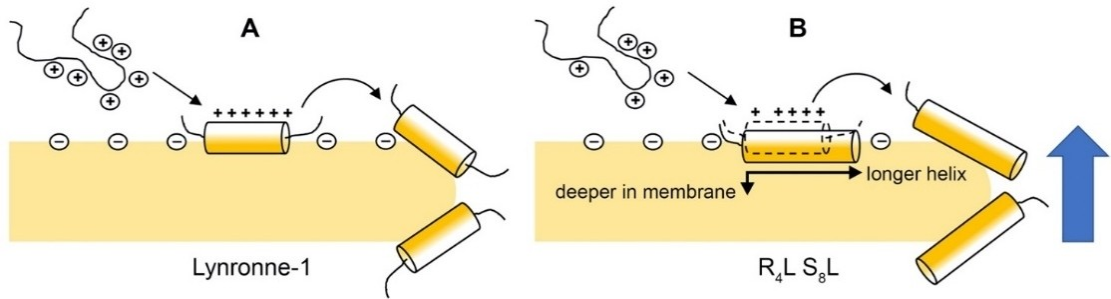
A. Model of Lynronne‐1 folding and function based on our biophysical data. From left to right, unfolded Lynronne‐1 selectively folds into an amphipathic α‐helix between residues 3–15 upon binding to membrane bilayers containing anionic PG lipids. Increased concentration of membrane‐bound Lynronne‐1 leads to lysis, which our data and the length of the helix suggest is via the carpet model, and this lysis is sensitive to lipid composition. B. Analogous model for R_4_L‐S_8_L based on our biophysical data (dashed lines indicate relative position of wild type Lynronne‐1 for reference), in which the unfolded R_4_L‐S_8_L selectively folds into an amphipathic α‐helix which is longer than that in Lynronne‐1, extending to the C‐terminus. We suggest that this peptide can penetrate more deeply into the bilayer and yielded more rapid lysis of model membranes *in vitro* as well as improved antimicrobial activity *in vivo*.

In this work, we carried out a combined biophysical and microbiological investigation of the recently identified antimicrobial peptide Lynronne‐1 from the bovine rumen microbiome,^[11]^ a peptide which showed promising activity against a wide range of pathogens. In aqueous conditions, Lynronne‐1 was unfolded and remained so in the presence of membranes mimicking mammalian cells, unable to bind to the membrane. With detergents and anionic (PG‐containing) lipid vesicles, Lynronne‐1 bound and folded into a strongly α‐helical structure between residues R_3_‐V_15_ (a region which contains all six cationic residues in the peptide). While this helical region is significantly longer than previously predicted, the lipid‐binding behaviour correlates well with previous work on Lynronne‐1 which showed that specific lipid components are required for the peptide to interact with membranes,^[11]^ and demonstrates a clear preference for anionic (PG) headgroups that would form favourable interactions with positively charged residues in the sequence (Figure 1A). Binding of Lynronne‐1 to anionic membranes lead to rapid lysis of model membranes, and the length of the APH as well as our *in vitro* lysis data in the presence of dextran point away from a pore‐formation model and towards a detergent/carpet model of activity for these antimicrobial peptides, although this remains to be confirmed in future studies. Penetration of the hydrophobic face of the APH in Lynronne‐1 into the hydrophobic interior of the bilayer would destabilise the bilayer in a dose‐dependent manner (as shown in Figure 4B), eventually leading to full lysis. A schematic summarizing our proposed model for Lynronne‐1 function is shown in Figure 6A.

Lynronne‐1 has broad‐spectrum activity despite the fact that it appears to have a selectivity filter: positively charged amino‐acid(s) placed near the centre of the non‐polar face of the amphipathic αhelix **(**Figure 3C). Such a selectivity filter has been shown previously to improve antimicrobial activity, reduce haemolytic activity, and massively improve therapeutic indices for gram negative bacteria when included in a broad‐spectrum AMP.^[31]^ Given that Lynronne‐1 already appears to contain such a filter and yet retained its broad‐spectrum activity, there was no scope in systematic modification along these lines. Therefore, instead of altering properties such as the net charge and length of the AMP or inclusion of a selectivity filter, we rationally designed a small library of peptide variants in which the size of the hydrophobic face/polar angle of the APH was varied (Figure 5A) while minimising changes to other physicochemical properties of the peptide (e. g. length, charge, mass, etc.). Previous work suggests that the size of the hydrophobic face affects the ability of the peptides to insert into the membrane via the ‘snorkel’ effect, where shallow hydrophobic faces have reduced antimicrobial activity.^[32]^ For example, increasing the size of the hydrophobic face of the antimicrobial peptide Gomesin allows for deeper penetration of model membranes, resulting in more leakage of carboxyfluorescein.^[33]^ Likewise, decreasing the polar angle in helical peptides has been shown to increase membrane permeability for model peptides^[28]^ and antimicrobial peptides.^[34]^ Our biophysical data for Lynronne‐1 were wholly consistent with these previous studies and suggested that α‐helicity and membrane binding/lysis increased as the size of the hydrophobic helical face was increased, with R_4_L S_8_L producing the most helical and most lytic peptide. A schematic summarizing our proposed model for R_4_L S_8_L function is shown in Figure 6B.

The R_4_L S_8_L peptide has lost one cationic residue, and thus has a net charge of +5 (as opposed to +6 in Lynronne‐1). Given that all six cationic residues are present in the amphipathic helix of Lynronne‐1, as shown from our NMR data, we suggest that the loss of a single cationic residue does not adversely impact activity (activity improves significantly) but may impact selectivity as evidenced by the higher haemolytic activity of this variant. Previously, peptide hydrophobicity has been observed to be correlated with haemolytic activity, as peptides with higher hydrophobicities are able to penetrate deeper into the hydrophobic core of the erythrocyte membrane, resulting in haemolysis.^[35]^ Our results are in complete agreement with this model, both *in vitro* in model membranes of varying composition and *in vivo* via antimicrobial screening of Lynronne‐1 and the three designed variants against *A. baumannii* and *S. aureus*. In these organisms, we see a direct relationship between antimicrobial activity and the size of the hydrophobic helical face. Thus, this simple strategy could be applied to any antimicrobial peptide using a minimal number of substitutions to gain an increase in activity without compromising selectivity.

## Experimental Section

**Peptide synthesis and purification**: 19‐residue peptides with sequences corresponding to wild‐type Lynronne‐1 (LPRRNRWSKIWKKVVTVFS), and three rationally designed variants I_10_T V_15_T, V_15_T, and R_4_L S_8_L, were synthesised using Fmoc chemistry and purified to 95 % purity at Insight Biotechnology Limited (Wembley, UK). Peptide purity was determined by HPLC and electrospray ionization time‐of‐flight mass spectroscopy (ESI‐TOF‐MS) by the manufacturer and further confirmed by matrix assisted laser desorption ionization time‐of‐flight mass spectrometry (MALDI‐TOF‐MS, Bruker Autoflex) upon delivery. See Figures S1–S4 for mass spectra of all peptides. All peptides were lyophilised and stored as dry powders at −20 °C until use.

**Model membrane preparation**: Detergent solutions were prepared from dodecyl phosphocholine (DPC), 1‐myristoyl‐2‐hydroxy‐sn‐glycero‐3‐phospho‐(1′‐rac‐glycerol) (LMPG), or *n*‐dodecyl‐β‐D‐maltoside (DDM). Vesicles with the variety of compositions detailed in Table 1 were prepared by dissolving lipids in a 3 : 1 chloroform:methanol solution to a concentration of 10 mg/mL, followed by drying to a thin film under vacuum on a rotary evaporator. Resultant lipid films were hydrated in 25 mM sodium phosphate buffer, pH 6.8, to a final concentration of 3 mg/mL. Four cycles of sonication, freezing at −20 °C and thawing at room temperature were then carried out to ensure vesicle formation and sizing. Where indicated, vesicles were extruded through a 50 nm pore diameter polycarbonate membrane, using a mini‐extruder (Avanti Polar Lipids), immediately prior to use. All lipids were supplied by Avanti Polar Lipids, Inc. (Alabaster, AL) and used without additional purification.

**Circular dichroism**: CD spectra were measured using a Jasco J‐1500 spectropolarimeter equipped with a Peltier thermally controlled cuvette holder (Jasco UK, Great Dunmow, UK) and a 1.0 mm path length quartz cuvette (Starna, Optiglass Ltd., Hainault, UK). All spectra were recorded over the wavelength range 190–300 nm using a 2.0 nm spectral bandwidth, 0.1 nm step resolution, 100 nm/min scanning speed, and 1 s response time. CD spectra shown were collected at 37 °C and were averaged from eight individual spectra after subtraction of the relevant peptide free blank solution CD spectrum. Peptides were dissolved in 25 mM sodium phosphate buffer, pH 6.8 at a maintained concentration of 50 μM across all measurements in the presence and absence of vesicles (1.5–2.5 mg/mL) or detergents (50 mM DPC, 50 mM DDM, and 14 mM LMPG). Peptide concentration was calculated from the observed absorbance at 280 nm (A_280_) and an estimated extinction coefficient of ϵ=11,000 M^−1^ cm^−1^ using the Beer‐Lambert law. CD data were converted from machine units (millidegrees) to mean residue ellipticity (MRE) before plotting.

**Nuclear magnetic resonance**: Peptide samples for NMR analyses were prepared by dissolving the peptide to a final concentration of 0.6–0.8 mM in 25 mM sodium phosphate buffer pH 6.8 containing 10 % D_2_O and 100 mM DPC‐*d_38_*. ^1^H‐^1^H TOCSY and NOESY spectra were collected at 37 °C with 2048×256 data points, 64–128 scans, and mixing times (τ_m_) ranging from 70–150 ms. All spectra were collected on an Avance 700 MHz spectrometer (Bruker Biospin, UK) equipped with a triple resonance cryoprobe with Z‐gradients and referenced to residual water. Spectra were processed and assigned using Sparky.^[36]^

Tryptophan fluorescence: Fluorescence spectra were measured using a Jasco FP‐6500 spectrophotometer (Jasco UK, Great Dunmow, UK) with a 1.0 mm path length quartz cuvette (Starna, Optiglass Ltd., Hainault, UK). The excitation wavelength was set to 295 nm and emission was recorded between 300 and 400 nm at room temperature with a bandwidth of 3 nm. Peptides were dissolved in 25 mM sodium phosphate buffer (pH 6.8) to a concentration of 5 μM, and measurements were made in the presence and absence of a variety of mammalian and bacterial model membranes at a concentration of 0.12 mg/mL.

*Membrane lysis assay*. A solution of 20 mM carboxyfluorescein was prepared by dissolving solid carboxyfluorescein in an appropriate volume of 25 mM sodium phosphate buffer followed by correction of pH to 6.8. Vesicles were prepared by solvation of lipids in a 3 : 1 chloroform:methanol mixture and dried under vacuum to produce a thin film. Lipids were hydrated in 20 mM carboxyfluorescein solution to a final lipid concentration of 3 mg/mL and subjected to four freeze‐thaw‐sonication cycles. Vesicles were extruded through a 100 nm polycarbonate membrane using a mini‐extruder (Avanti Polar Lipids). Immediately following extrusion, carboxyfluorescein‐containing vesicles were separated from free fluorophore using size exclusion chromatography on a Sephadex® G‐25 column with 25 mM Sodium phosphate buffer (pH 6.8) as the mobile phase. Purified vesicles were diluted 100‐fold to achieve a change in fluorescence intensity within the detection range of the fluorimeter (Jasco FP‐6500, Jasco UK, Great Dunmow, UK). The excitation wavelength was set to 492 nm and fluorescence emission intensity was recorded at 512 nm in 1 second intervals for 800 seconds after addition of peptides. Data was normalised to the basal fluorescence intensity prior to peptide addition and to a 100 % disruption value obtained by addition of 0.1 % by volume Triton‐X100 detergent. Peptides were added from concentrated stocks dissolved in 25 mM sodium phosphate buffer (pH 6.8).

**Dynamic light scattering**: Dynamic light scattering (DLS) was used to confirm the homogeneity and hydrodynamic diameter of vesicles after extrusion and upon addition of peptides in samples containing lipid concentrations of 0.05 mg/mL in 25 mM sodium phosphate buffer (pH 6.8). The measurements were performed on the Zetasizer Nano‐series instrument (Malvern Instruments, UK) at room temperature, with UV‐transparent disposable cuvettes of 1 cm path length. All DLS spectra were collected after 300 s equilibration time. Data was processed using the Malvern Zetasizer software and exported as number distributions.

**Activity measurements**: MICs were determined using a broth microdilution method^[37]^ in cation‐adjusted Mueller‐Hinton broth. Peptides were dissolved in sterile distilled water in low‐binding polypropylene Eppendorf tubes, and their concentration was calculated from the observed absorbance at 280 nm (A_280_) and an estimated extinction coefficient of ϵ=11,000 M^−1^ cm^−1^ using the Beer‐Lambert law. Peptide solutions and quality control antibiotics (carbenicillin and ciprofloxacin) dissolved in appropriate solvents^[38]^ were added to sterile flat‐bottom polystyrene 96‐well microplates at concentrations of approximately 0.5–256 μg/mL. The final concentration of bacterial inoculum in each well was 7.5×10^5^ CFU/mL. MIC was defined as the lowest concentration of peptide or antibiotic which inhibited the visible growth of bacteria after 18–20 hours at 37 °C. A total of three biological repeats were carried out to confirm activity. Haemolytic activity was determined for all antimicrobial peptides.^[39]^ Equine blood was centrifuged at 1,000×g for 10 min and the supernatant and the buffy coat were removed. The erythrocytes were washed three times with cold 0.9 % saline solution and resuspended to a concentration of 5 % in 0.9 % saline. Peptide solutions were added to sterile round‐bottom polystyrene 96‐well microplates at concentrations of approximately 2–2000 μg/mL. Controls of 0.9 % saline and 5 % Triton X‐100 were used for 0 % and 100 % haemolysis respectively. Erythrocytes were then added to each well and incubated at 37 °C for one hour. Lack of haemolysis was noted by formation of a ‘button’ of erythrocytes in the bottom of the well.

## Conflict of interest

The authors declare no conflict of interest.

## Supporting information

As a service to our authors and readers, this journal provides supporting information supplied by the authors. Such materials are peer reviewed and may be re‐organized for online delivery, but are not copy‐edited or typeset. Technical support issues arising from supporting information (other than missing files) should be addressed to the authors.

Supplementary
