# Supplementary material for: Molecular Basis of Selectivity and Activity for the Antimicrobial Peptide Lynronne‐1 Informs Rational Design of Peptide with Improved Activity
Source: Chembiochem. 2021 Jun 8;22(14):2430–9. doi: 10.1002/cbic.202100151 (PMC8362026; doi:10.1002/cbic.202100151)
Supplement: Supplementary file 1 — Supplementary [file CBIC-22-2430-s001.pdf]

ChemBioChem

Supporting Information

Molecular Basis of Selectivity and Activity for the Antimicrobial Peptide Lynronne-1 Informs Rational Design of Peptide with Improved Activity

Eleanor S. Jayawant, Jack Hutchinson, Dorota Gašparíková, Christine Lockey, Lidón Pruñonosa Lara, Ciaran Guy, Rhiannon L. Brooks, and Ann M. Dixon*

Supporting Information

Table S1. ^{1}H-NMR chemical shifts for wild-type Lynronne-1 (0.6 mM, $C_{113}H_{182}N_{34}O_{24}$) dissolved in 25 mM sodium phosphate buffer, pH 6.8, in the presence of 100 mM DPC-d_{38}.

Residue	NH	αH	βH	Others
**Leu1**	7.97	3.42	1.74	γCH *unassigned*<br>δCH_3 0.61, 0.46
**Pro2**	n/a	4.51	2.34	γCH_2 1.98, 1.82<br>δCH_2 3.79, 3.50
**Arg3**	7.91	4.04	1.99	γCH_2 1.82<br>δCH_2 2.18<br>scNH *unassigned*
**Arg4**	7.89	3.81	1.91, 1.66	γCH_2 1.47<br>δCH_2 2.97<br>scNH 7.71, 6.98
**Asn5**	8.06	4.59	2.86, 2.82	γNH_2 *unassigned*
**Arg6**	8.53	3.99	1.75	γCH_2 1.53<br>δCH_2 3.18, 3.10<br>scNH 7.43, 6.92
**Trp7**	8.40	4.59	2.82	NH' 10.65<br>2H 7.35<br>4H 7.02<br>5H 7.37<br>6H 6.88<br>7H 7.56
**Ser8**	8.29	4.24	3.99	n/a
**Lys9**	7.82	4.10	1.90	γCH_2 1.46<br>δ$CH_2$1.67,1.59<br>εCH_2 2.96<br>εNH_3 *unassigned*
**Ile10**	8.06	3.51	2.11	γCH_3 1.05<br>δCH_3 *unassigned*
**Trp11**	8.51	4.45	3.86	NH' 10.48<br>2H 7.19<br>4H 7.11<br>5H 7.35<br>6H 6.97<br>7H 7.52
**Lys12**	8.64	4.24	1.79	γCH_2 0.96<br>δ$CH_2$1.66<br>εCH_2 3.19<br>εNH_3 *unassigned*
**Lys13**	7.51	4.10	1.99	γCH_2 1.48<br>δ$CH_2$1.67<br>εCH_2 2.85<br>εNH_3 *unassigned*
**Val14**	7.85	3.74	2.13	γCH_3 1.00
**Val15**	7.78	3.81	*unassigned*	γCH_3 0.91
**Thr16**	7.57	4.29	3.98	γCH_3 1.26

**Val17**	7.63	3.68	2.01	γCH_3 0.86, 0.42
**Phe18**	7.30	4.55	3.39, 2.71	2,6 H 7.11<br>3,5 H *unassigned*<br>4H *unassigned*
**Ser19**	7.36	4.30	3.87, 2.97	n/a

Table S2. ^{1}H-NMR chemical shifts for Lynronne-1 R_4L S_8L mutant (0.8 mM, $C_{116}H_{187}N_{31}O_{23}$) dissolved in 25 mM sodium phosphate buffer, pH 6.8, in the presence of 100 mM DPC-d_{38}.

Residue	NH	αH	βH	Others
**Leu1**	*unassigned*	*unassigned*	*unassigned*	γCH *unassigned*<br>δCH_3 *unassigned*
**Pro2**	n/a	4.42	2.28, 1.90	γCH_2 1.87, 1.74<br>δCH_2 3.74, 3.42
**Arg3**	8.59	4.12	1.69, 1.61	γCH_2 1.51<br>δCH_2 3.11<br>scNH 7.31, 6.94
**Leu4**	8.39	4.11	1.59	γCH *unassigned*<br>δCH_3 0.83, 0.77
**Asn5**	8.15	4.30	2.77, 2.68	γNH_2 7.70, 6.91
**Arg6**	8.14	3.95	1.70	γCH_2 1.28<br>δCH_2 2.78<br>scNH 6.87, 6.62
**Trp7**	7.81	4.46	3.31	NH' 10.42<br>2H 7.15<br>4H 7.30<br>5H 6.94<br>6H 6.83<br>7H 7.38
**Leu8**	8.11	4.16	1.33	γCH *unassigned*<br>δCH_3 0.94, 0.87
**Lys9**	7.72	3.97	1.88	γCH_2 1.36<br>δCH_2 1.59<br>εCH_2 2.88<br>εNH_3 6.99
**Ile10**	7.61	3.70	2.13	γCH_3 1.45<br>δCH_3 0.92
**Trp11**	8.41	4.37	3.34	NH' 10.27<br>2H 7.00<br>4H 7.22<br>5H 6.87<br>6H 6.79<br>7H 7.32
**Lys12**	7.45	4.03	1.93	γCH_2 1.42<br>δ$CH_2$1.59<br>εCH_2 2.78<br>εNH_3 7.44
**Lys13**	8.03	3.66	1.82	γCH_2 1.39<br>δ$CH_2$1.59<br>εCH_2 2.87<br>εNH_3 7.29
**Val14**	7.86	3.35	1.60	γCH_3 0.51, 0.36
**Val15**	8.03	3.45	2.09	γCH_3 0.98, 0.79
**Thr16**	7.42	4.21	3.92	γCH_3 1.19

**Val17**	7.54	3.64	1.95	γCH_3 0.76, 0.38
**Phe18**	7.23	4.48	3.33, 2.65	2,6 H 7.05<br>3,5 H 7.26<br>4H 6.95
**Ser19**	7.28	4.21	3.79	n/a

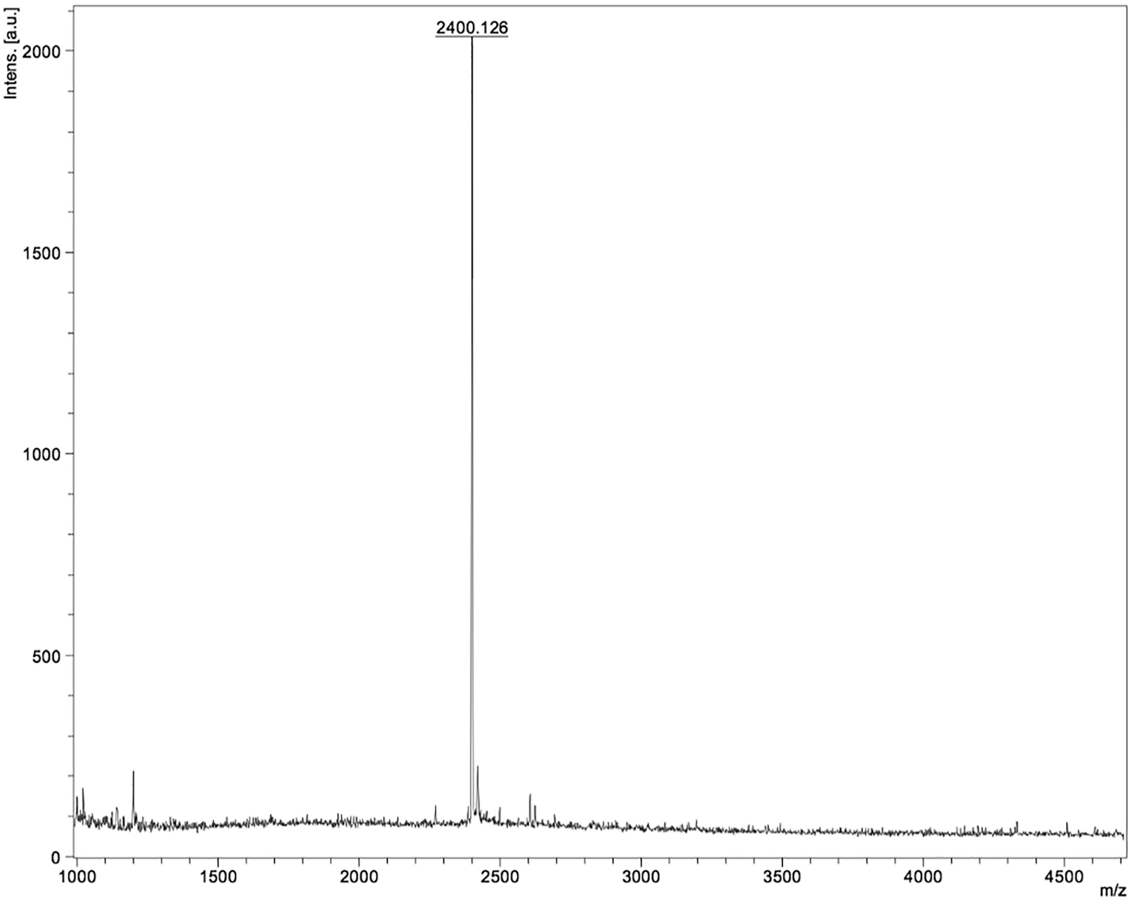

Figure S1: MALDI TOF spectrum of Lynronne-1 (MW 2400.9 Da) collected in the matrix 3,5-Dimethoxy-4-hydroxycinnamic acid (i.e. Sinapic acid).

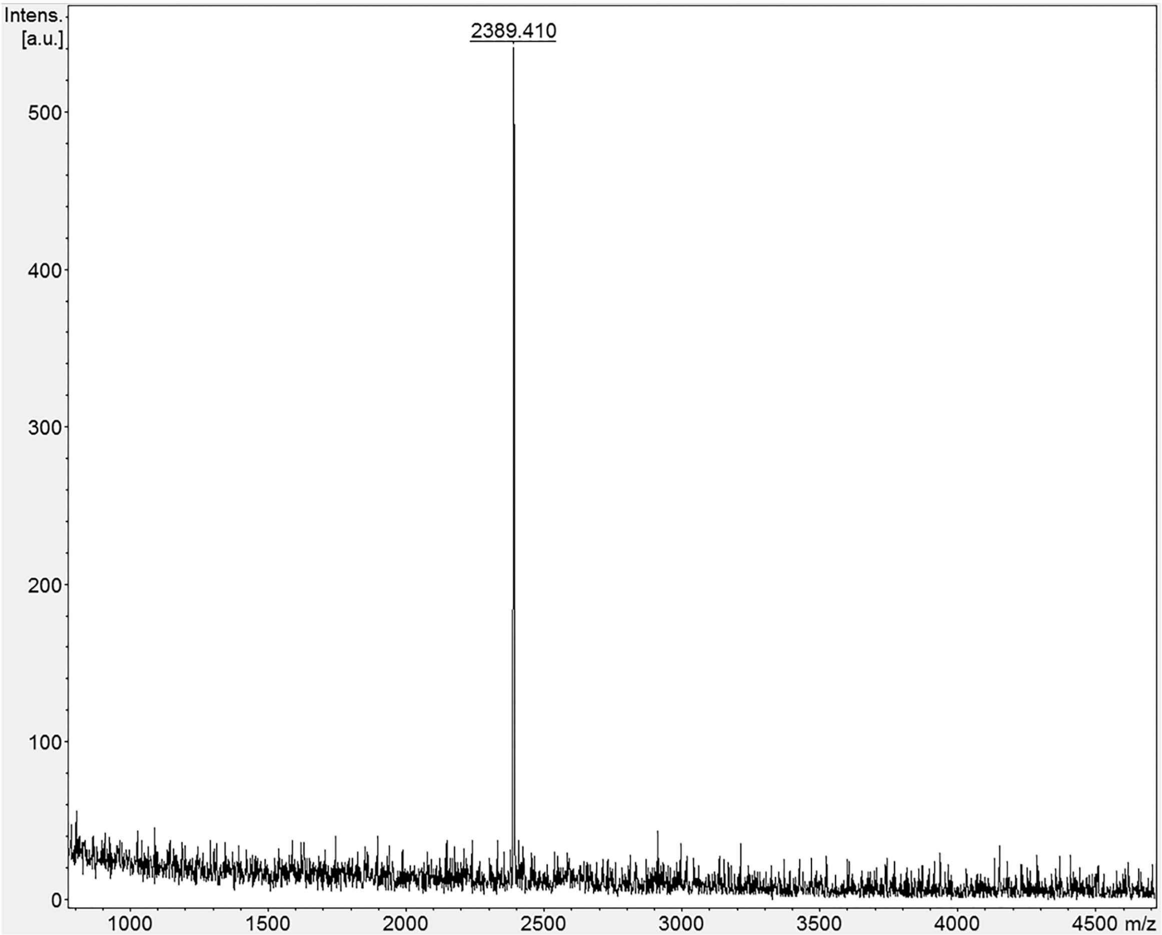

Figure S2: MALDI TOF spectrum of $I_{10}T$ $V_{15}T$ (MW 2390.8 Da) collected in the matrix 3,5-Dimethoxy-4-hydroxycinnamic acid (i.e. Sinapic acid).

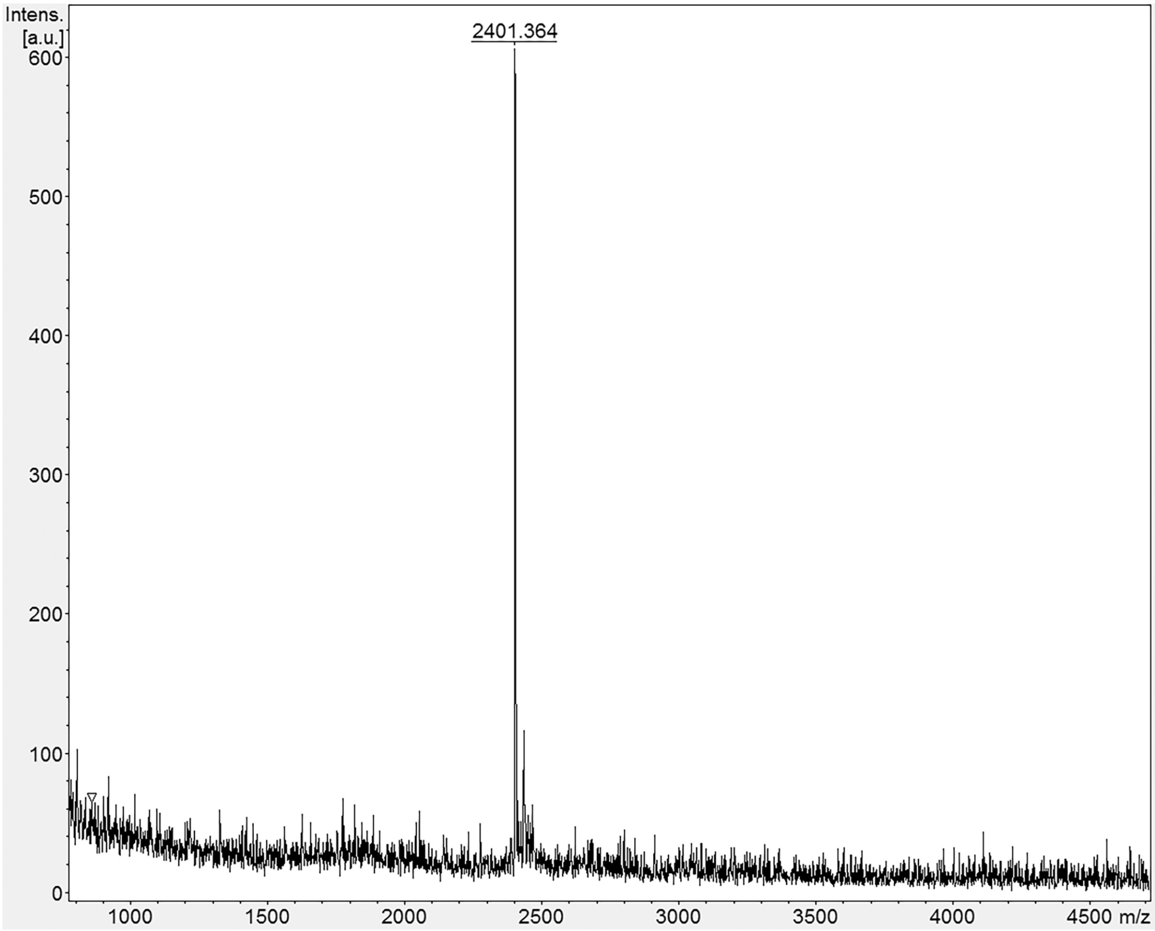

Figure S3: MALDI TOF spectrum of $V_{15}T$ (MW 2402.9 Da) collected in the matrix 3,5-Dimethoxy-4-hydroxycinnamic acid (i.e. Sinapic acid).

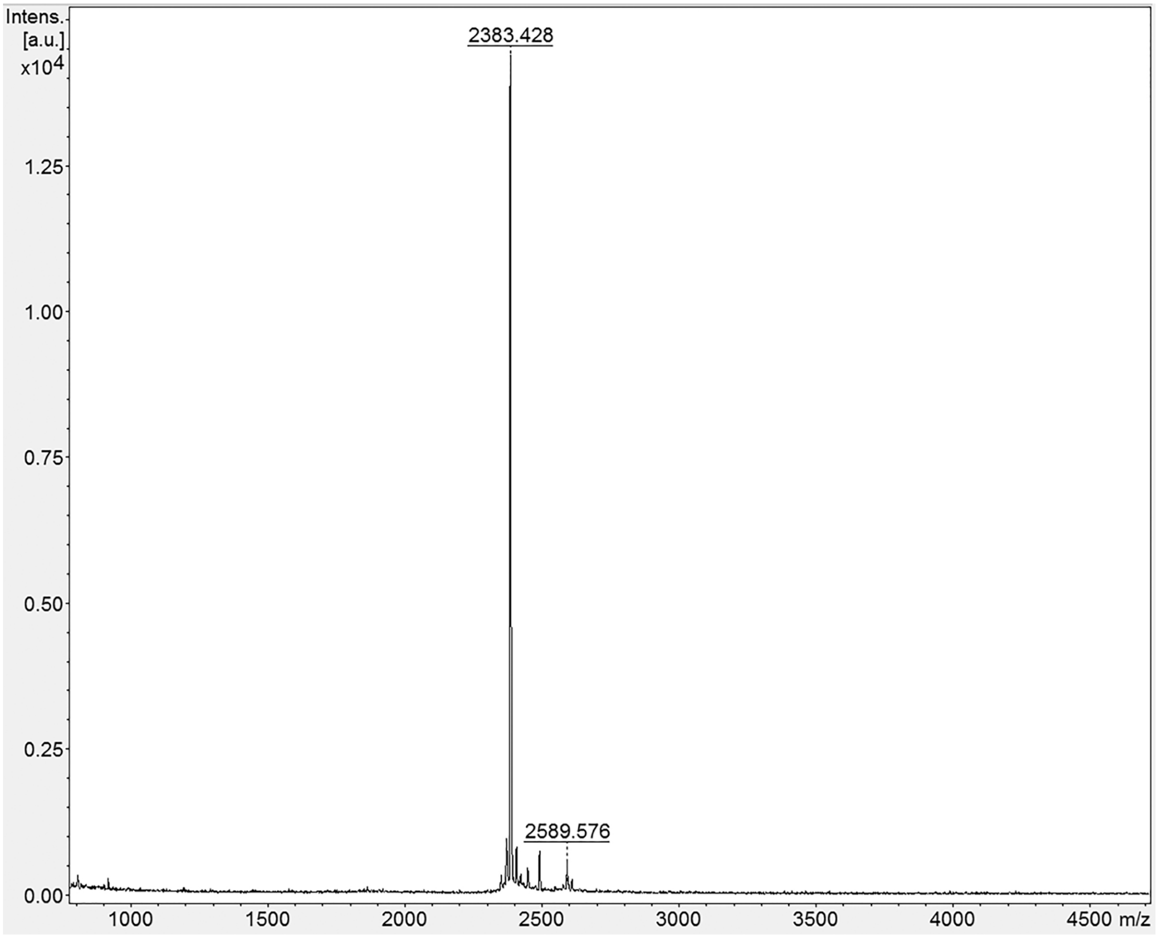

Figure S4: MALDI TOF spectrum of R_4L S_8L (MW 2383.95 Da) collected in the matrix 3,5-Dimethoxy-4-hydroxycinnamic acid (i.e. Sinapic acid).

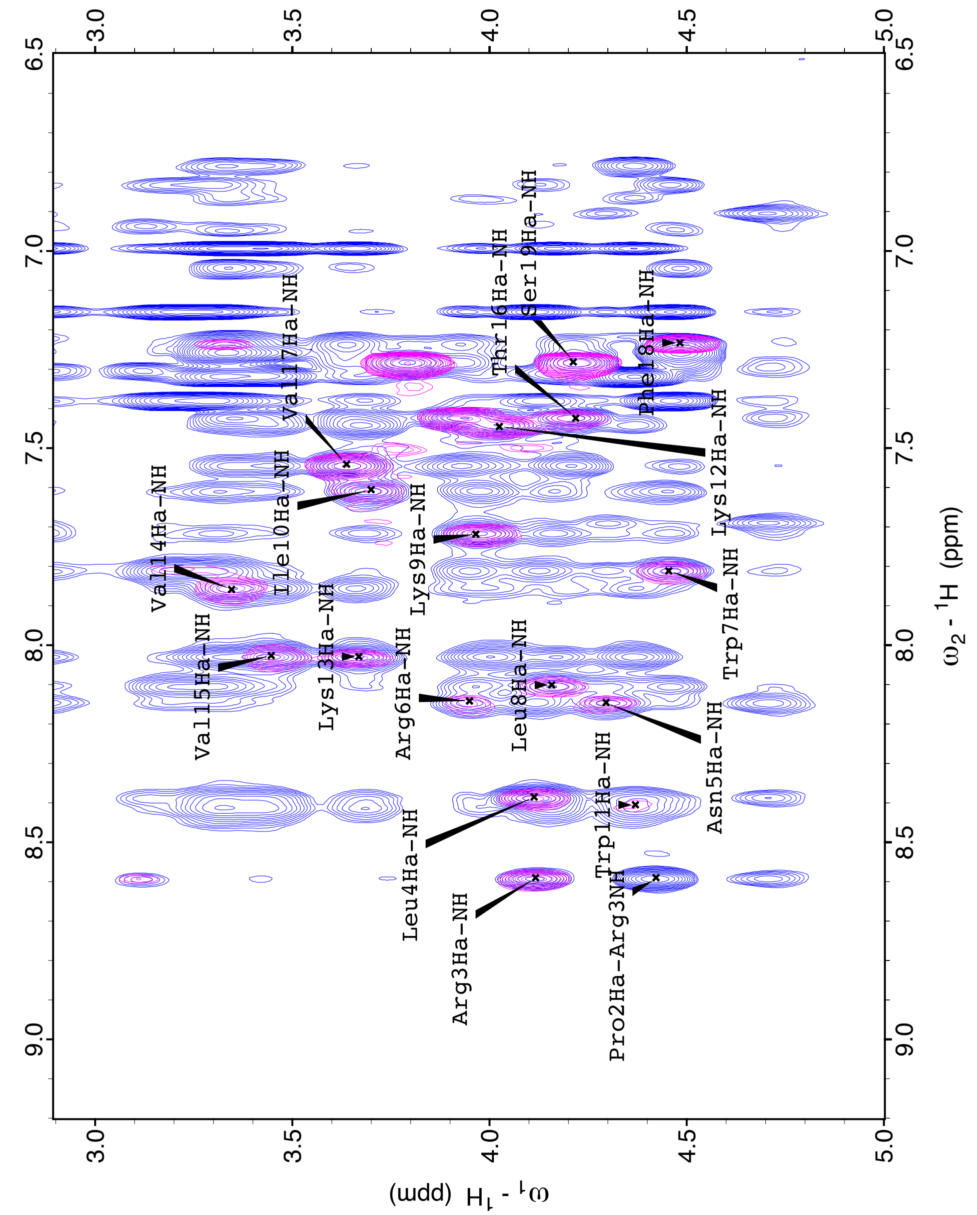

Figure S5: Overlay of ^{1}H-^{1}H NOESY (τ_m=150 ms, blue spectrum) and ^{1}H-^{1}H TOCSY (τ_m=100 ms, pink spectrum) showing the fingerprint region of the R_4L S_8L peptide (0.8 mM) in 25 mM sodium phosphate buffer, pH 6.8, containing 100 mM DPC-*d*$_{38}$. The Hα-NH correlations required to perform a backbone-walk have been labelled.

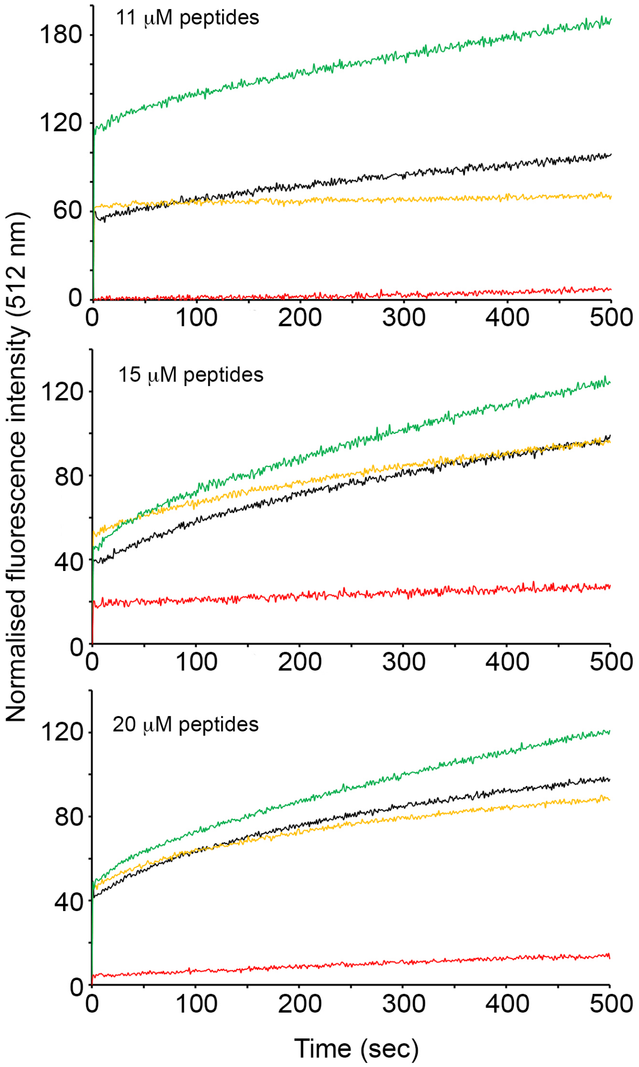

Figure S6: Fluorescence time course measurements collected over 500 seconds at 512 nm for carboxyfluorescein-loaded 8:2 POPC:POPG vesicles in the presence of wild-type Lynronne-1 and three variants. Data were normalized to Lynronne-1 (black line, set to 100 at 500 sec), and plotted for $V_{15}T$ (amber line), $I_{10}T$-$V_{15}T$ (red line) and R_4L-S_8L (green line). Measurements were made at three peptide concentrations: 11 μM (top panel); 15 μM (middle panel); and 20 μM (bottom panel). The observed trend in lytic activity was R_4L-S_8L > Lynronne-1 = $V_{15}T$ > $I_{10}T$-$V_{15}T$ and was identical in all three data sets.
